# Respiratory droplets interception in fibrous porous media

**DOI:** 10.1063/5.0060947

**Published:** 2021-08-05

**Authors:** Dario Maggiolo, Srdjan Sasic

**Affiliations:** Department of Mechanics and Maritime Sciences, Chalmers University of Technology, Göteborg SE-412 96, Sweden

## Abstract

We investigate, by means of pore-scale lattice Boltzmann simulations, the mechanisms of interception of respiratory droplets within fibrous porous media composing face masks. We simulate the dynamics, coalescence, and collection of droplets of the size comparable with the fiber and pore size in typical fluid-dynamic conditions that represent common expiratory events. We discern the fibrous microstructure into three categories of pores: small, large, and medium-sized pores, where we find that within the latter, the incoming droplets tend to be more likely intercepted. The size of the medium-sized pores relative to the fiber size is placed between the droplet-to-fiber size ratio and a porosity-dependent microstructural parameter $L_{\epsilon }^{*}=\epsilon /(1-\epsilon )$, with *ϵ* being the porosity. In larger pores, droplets collection is instead inhibited by the small pore-throat-to-fiber size ratio that characterizes the pore perimeter, limiting their access. The efficiency of the fibrous media in intercepting droplets without compromising breathability, for a given droplet-to-fiber size ratio, can be estimated by knowing the parameter $L_{\epsilon }^{*}$. We propose a simple model that predicts the average penetration of droplets into the fibrous media, showing a sublinear growth with $L_{\epsilon }^{*}$. Permeability is shown also to scale well with $L_{\epsilon }^{*}$ but following a superlinear growth, which indicates the possibility of increasing the medium permeability at a little cost in terms of interception efficiency for high values of porosity. As a general design guideline, the results also suggest that a fibrous layer thickness relative to the fiber size should exceed the value $L_{\epsilon }^{*}$ in order to ensure effective droplets filtration.

## I. INTRODUCTION

Transport of particles and emulsions in porous media is of great interest in numerous applications such as oil recovery,^1^ remediation of groundwater,^2^ oil-mist filtration,^3^ energy,^4^ and many more. In all these examples, one can look at particles of sizes much smaller than the fiber and pore size,^5^ at those with sizes comparable to that of the mean pore size,^6^ but also at those that are much bigger than the pores.^7^ In the latter case, there will be a squeezing process that requires significant drop deformation and hydrodynamic resistance. We are in this work interested in the case where droplets are transported through a porous medium made of fibers of the similar size as the droplets, and for which the expected dominant mechanism of collection in the porous medium is given by the collision of droplets with fibers found along their trajectories, a mechanism referred to as interception. Such a study is beneficial in many applications, but we are here looking at the behavior of pathogen-bearing droplets in face masks, a problem that is of great relevance in the current pandemic.

Acute respiratory virus illnesses comprise a wide range of symptoms caused by respiratory virus infections. While most often mild, these illnesses sometimes appear in more severe forms, causing wide-range infections and even death.^8^ The current COVID-19 pandemic well represents a devastating range of effects that such diseases can have on modern societies. An enormous body of literature has already been created and will certainly be created even more in the future, on various medical, economical, socio-psychological, and many other aspects of the current pandemic.

In the course of COVID-19 pandemic, different countries have formulated rather different precautionary and protective measures. Very often, there is a lack of consensus on the effectiveness of a certain measure, and the question of wearing face masks is a good example in that manner. At least in the early months of the pandemic, there have been conflicting reports on whether masks represent an effective physical intervention against transmission of the disease.^9^ Discussions related to the use of masks comprise questions like the effectiveness of masks, their availability in the early stage of the pandemic, perceptions of masks in different cultural environments, formulations of mask-use strategies, and similar. Recommendations varied from masks to be worn only by infected individuals to prevent onward transition (the so-called source control strategy^10^) to wearing masks as a must for the general population, especially indoors. A need was, thus, promptly recognized^11^ to provide policy makers with guidelines on the use of masks by general population as a tool in opposing COVID-19. It is now widely accepted^12^ that masks should have a synergetic effect with other measures, i.e., that they are a complement rather than an alternative to other health control measures. In other words, a face mask cannot provide complete protection but should be used in conjunction with, for example, social distancing recommendations.

Given the importance of acute respiratory virus diseases even before the COVID-19 pandemic, it is surprising how relatively little attention face masks have previously received as a possible protective measure. The research is, however, now advancing rapidly and comprises a wide array of aspects such as mask efficiency, virus transmission characteristics, wearer protection, and implementation- and sociological considerations. Significant progress has indeed been achieved, but uncertainties remain on the modes of transmission of COVID-19^13^ and from the fact that most of the evidence on the filtering efficiency of face masks comes from *in vitro* experiments with non-biological particles.^14^ Knowledge is needed and acquired on different phenomena that potentially influence the performance of a face mask: nature of expiratory events,^15^ properties of respiratory droplets,^16^ effects of design of face masks (e.g., related to the importance of leakages^17^), and fundamental mechanisms that form the principles of face mask functioning.^18,19^

World Health Organization identifies two main categories of particles as key factors in virus transmission: respiratory droplets (larger than 10 *μ*m) and droplet nuclei (aerosols that are smaller than 5 m), and there are still open questions about the relative importance of these modes of transmission.^20^ Depending on the size of pathogen-bearing particles, the following mechanisms are identified^18^ behind their trapping in a face mask: diffusion, electrostatic attraction, and inertial and direct interception. It is interesting that for a given filter, there is a pathogen size for which the filtration efficiency is at minimum.^17^ The first two mechanisms identified above dominate for small pathogens (relative to the size at which the minimum efficiency is identified), whereas interception prevails for large pathogen-bearing droplets.

There exist a number of considerations related to mask performance: efficiency, breathability, fit, and continued performance under operating conditions (decrease in efficiency with time). A mask is to be designed in such a way to pass those tests and to meet sometimes conflicting requirements. In general, face masks are designed with multiple layers. These layers are fibrous porous media composed of fibers with diameters of the order of a few micrometers and with variable orientations. The layers are not only responsible for most of the particle removal but also for flow resistance. If the thickness of a layer is increased, a higher filtering efficiency is achieved, but followed with consequent difficulties in breathing and a significant decrease in wearing comfort.

There exist numerous both experimental and numerical studies that evaluate the performance of face masks, and not all of them were carried out during the COVID-19 pandemic. An experimental study looked at the behavior of a forward jet of droplets from a mannequin model using the Schlieren optical method and identified regions of leakage from a mask.^21^ Another work investigated the air dispersion distances during coughing of a human patient simulator, both without and with different types of masks (surgical and N95 masks).^22^ The same study showed that a N95 mask prevented air leakage more effectively than did a surgical mask, although significant sideway leakage was recognized even with the former mask. The effect on the efficiency of a mask of gaps between the contour of a face and the mask is a common subject in experimental studies.^23^ Other experimental studies pointed out that it is difficult to mimic truly correct wearing conditions, since human skin surface is soft, while the manikins have a hard (solid) surface.^17^ Recent experimental studies used particle image velocimetry to look at the performance of various masks in cases where social distancing is not possible and identified a minimum distance (around 6 ft) below which masks would not offer complete protection from airborne sneeze and cough droplets.^24^ Another study used visualization techniques to investigate how material- and design-choices impact the extent to which droplet-laden respiratory jets are blocked.^25^ The authors concluded that well-fitted masks with multiple layers of quilting fabric proved to be the most effective in reducing droplet dispersal. A similar study examined the effects of using alternative protection means (face shields and masks with exhalation valves) and concluded that using such equipment significantly reduces the protection against virus-berating respiratory droplets.^26^ A recent review shed more light on important design aspects of face masks and their impact on overall comfort of a user (thermal comfort and breathability).^27^

Numerical simulations may provide a valuable tool for overcoming the limitations related to experimental techniques, either in an ability to study specific phenomena to look at or in terms of investment- and temporal costs.^28^ Numerical studies involving face masks are relatively less present than those that look at transport and residence time of expiratory droplets in open environments. In Ref. 29, the authors formulate a mathematical model for estimating the risk of airborne transmission of respiratory infections and use the model to assess the protection afforded by a variety of face protective equipment. There is a number of recent high-fidelity numerical studies of various expiratory events, both with and without face masks used.^30,31^ A range of phenomena have raised interest, from the mechanisms the droplets are dispersed,^32^ specific places where the spreading of infection may be of extra concern,^33^ probabilities of virus transmission in different situations,^34–36^ and also the effects on dispersion of respiratory droplets of human physiological factors that can be associated with illness, anatomy, stress condition, and similar.^37^ Dbouk and Drikakis^38^ performed their simulations using an Eulerian–Lagrangian multiphase framework and took into account dynamics of coughing, droplet breakup, evaporation, and the turbulent dispersion force. The presence of a face mask is reproduced by droplets impacting a porous structure of a thickness that represents the thickness of the mask filter. The porosity of the mask is described with the resistance to fluid flow, formulated as a pressure drop across the porous structure. The pressure drop term had viscous and inertial contributions with specific coefficients estimated from experimental studies.^39^ As functions of the Weber number and droplet diameter, the authors formulated stick, splash/rebound, and penetrate as modes of interaction of droplets and the mask structure. In Ref. 40, the authors formulated a mask-face-airway model, in which the viscous resistance of the mask in the normal direction was formulated from Darcy's law and using an overall mask thickness without differentiating multiple layers that typically comprise a face mask. The study focused on the likelihood of inhaling droplets when a mask is worn and presented penetration rates of droplets into lungs. The authors assumed a perfect seal between the mask and the face, a condition that is almost never fulfilled in practice due to various incorrect wearing practices.^41^

In this paper, we also do numerical simulations but adopt a different approach when looking at transport processes in fibrous porous media from that in Refs. 38 and 40. We do pore-scale direct numerical simulations using a lattice Boltzmann framework in order to elucidate the effects of fibrous microstructures on the behavior of droplets that are of size comparable to that of pores. The mechanisms of transport of such droplets in porous media are primarily mechanical interception and then capillary transport, and in this work, we will deal with the former. To understand the interception, we need to be able to predict the average distance traveled by droplets before being captured and the penetration length that takes place in different fibrous layers. The optimal fibrous layer design that improves mechanical filtration is difficult to know *a priori* given the complex physical mechanisms that determine the fluid transport and spatial distribution in liquid–gas flows in porous media. The efficiency of a filtering medium is a complex function of the droplet- and fiber-size-based capillary number and the system microstructural characteristics such as the droplet-to-fiber size ratio and contact angle. We will focus here on the role of fiber and pore sizes rather than on effects of different wettabilities of fibrous structures. The goal is to understand and quantify the efficiency of fibrous layers in intercepting incoming droplets and, thus, to suggest an optimal design, while not compromising comfort of a wearer (breathability). We carry out the simulations using a number of sample domains that are designed in such a way as to mimic anisotropy of fiber orientation present in mask fibrous layers. As a first step, we assume our droplets being a Newtonian fluid, although there is some evidence^42^ that saliva under some circumstances exhibits strong non-Newtonian features.

Note that we do not claim that we work with complete structures of face masks since, as indicated above, the latter typically consist of multiple layers. Instead, we look at the fundamental mechanisms of droplet interception in homogeneous fibrous layers. We will pay special attention to calculation of probabilities for pores of different sizes being invaded by the incoming droplets. We will show that this analysis leads to some counterintuitive results since, for example, large pores are found to be less likely to be invaded by droplets. Finally, our study will result in the formulation of a model for droplet interception and capturing in fibrous layers and the estimation of their efficiency.

## II. NUMERICAL METHODOLOGY

We make use of the lattice Boltzmann methodology (LBM) for simulating droplet transport through a set of artificially generated fibrous layers, to mimic and represent mask fibrous layers composed of variously oriented fibers. The LBM is well suited for simulations of two-phase flows in complex geometries, such as in fibrous layers,^43^ given its intrinsic computational efficiency that allows to reach pore-scale resolution at a limited computational cost.^44^ We use the open-source code lbdm;^45^ a series of validation test cases of the two-phase flow algorithm are found in our previous works.^46,47^ The streaming-collision computation is performed according to

$$\begin{array}{c} f_{\xi }(x+c_{\xi }\delta t,t+\delta t)-f_{\xi }(x,t)=-\tau^{-1}(f_{\xi }(x,t)-f_{\xi }^{eq}(\rho ,u))+F_{\xi }, \end{array}$$ (1)

where *ξ* indicates the lattice direction within the three-dimensional grid (D3Q19), x=(x,y,z) is the position vector, *t* is the simulation time, *τ* is the relaxation time, and $c_{\xi }$ is the discrete lattice-Boltzmann speed. The kinematic viscosity for both phases is $\nu =c_{s}^{2}(\tau -0.5)$, where *c_s_* is the speed of sound. The equilibrium velocity $\rho u_{eq}=\rho u+(\tau -1/2)F_{\rho }$ is used to compute the equilibrium distribution function $f_{\xi }^{eq}$, after having defined the fluid density *ρ* and momentum ρu via the statistical averaging of the distribution functions^48^

$$\rho =\sum_{\xi }f_{\xi }(x,t),$$ (2)

$$\rho u=\sum_{\xi }f_{\xi }(x,t)c_{\xi }+\frac{1}{2}\frac{\Delta p}{L}+\frac{1}{2}F_{\rho }.$$ (3)

In Eq. (1), the term $F_{\xi }\propto \Delta p/L$ mimics a pressure gradient Δp/L through the application of a body force acting along the streamwise direction, where we follow Guo's forcing scheme.^49^

To represent surface tension forces, we apply the Shan–Chen approach,^50^ mimicking the intermolecular van der Waals interactions through the computation of the density-dependent pseudopotential function $\Psi (\rho )=1-e^{-\rho }$ with the intermolecular force $F_{\rho }$ expressed as

$$F_{\rho }(x,t)=-G\Psi (x,t)\sum_{\xi }w_{\xi }\Psi (x+c_{\xi },t)c_{\xi }.$$ (4)

Here, $w_{\xi }$ represents the lattice weighting coefficient along the *ξ*th direction and G=−5.5 is the interaction strength. The separation of phases is mathematically formulated by the non-ideal equation of state $P(\rho )=\rho c_{s}^{2}+G/2\;c_{s}^{2}\Psi {\left(\rho \right)}^{2}$. Different approaches can be used to track the interface across the resulting smooth interface between the separated phases, such as thresholding or more accurate resolution-independent methods.^51,52^ Here, we use a thresholding method to track the interface, where we discern the two phases by defining the interface density $1/2(\rho_{1}+\rho_{2})$. Density values that fall above or below such a value are considered to belong to the more or less viscous phase, respectively.

Finally, at the fluid–fluid–solid three phase contact line, we perform a spatial averaging of the neighbor densities^53^

$$\rho (x_{cl},t)=N^{-1}\sum_{n=0}^{N}\rho (x_{n},t)+\delta_{w},$$ (5)

where $\rho (x_{cl},t)$ represents a fictitious density value at the solid node $x_{cl}$ near the contact line and n=0,…,N are the nearest fluid nodes. Given the great variability of wetting properties that the fibrous layer can exhibit, we choose to focus our analysis on the microstructural effects and restrict the simulations to neutrally wetted media with an equilibrium contact angle θ ∼90° ($\delta_{w}=0$).

### A. Fibrous microstructure

To mimic a microstructure of a layer composing face masks, we generate different fibrous porous media samples, by placing fibers with a random uniform distribution in a three-dimensional triperiodic box whose volume is V=Ah, where *A* is the cross-sectional area and *h* is the through-plane thickness along the streamwise direction *x*. With the chosen parameter space for our simulations, we specifically target the thickness of such layers and the fiber size,^54–56^ porosity of the medium,^55,56^ and the fiber orientation distribution (FOD) of some of the commonly used types of face masks. FOD is one of the key parameters that affect transport phenomena in a fibrous microstructure,^57^ and it can be obtained by having in mind that layers of a face mask are typically manufactured of non-woven fabrics (e.g., melt-blown non-woven polypropylene).^54,58^ In such structures, FOD can be obtained by x-ray computed tomography, where a range of azimuthal and polar angles is provided.^59^ We, thus, assign the fibers a random orientation within the intervals defined by the azimuthal *β* and polar ϕ angles, [45,90] and [0,360], respectively, which indicate a medium with fibers preferentially oriented along the transverse directions *y* and *z*. Such a preferential orientation should mimic the typical anisotropy encountered in thin fibrous layers such as the one composing face masks. These thin layers are indeed anisotropic not only according to their main fiber orientations but also because of the separation of scales that characterizes their through-plane and in-plane dimensions with the former being much smaller than the latter. An important parameter that characterizes such thin media is, thus, the ratio between the thickness *h* (100–500 μm) and the fiber size *d_f_* (5–10 μm), which is typically of the order of $h^{*}=h/d_{f}\;∼10–100$, while the in-plane dimension is a couple of orders of larger magnitude. We vary the porosity *ϵ* and fiber size for different samples to accommodate values that span between the typical ranges for face masks' fibrous layers.^55^ The ratio between in-plane and fiber dimensions is $\sqrt{A}/d_{f}=22.5-45$, a range of values that should guarantee homogeneity along the in-plane directions and a sufficient representation of the pore size distribution of the fibrous medium. Since we impose periodic boundary conditions along the in-plane directions, we aim at representing a medium infinitely long along such directions, *y* and *z*.

**FIG. 1.**
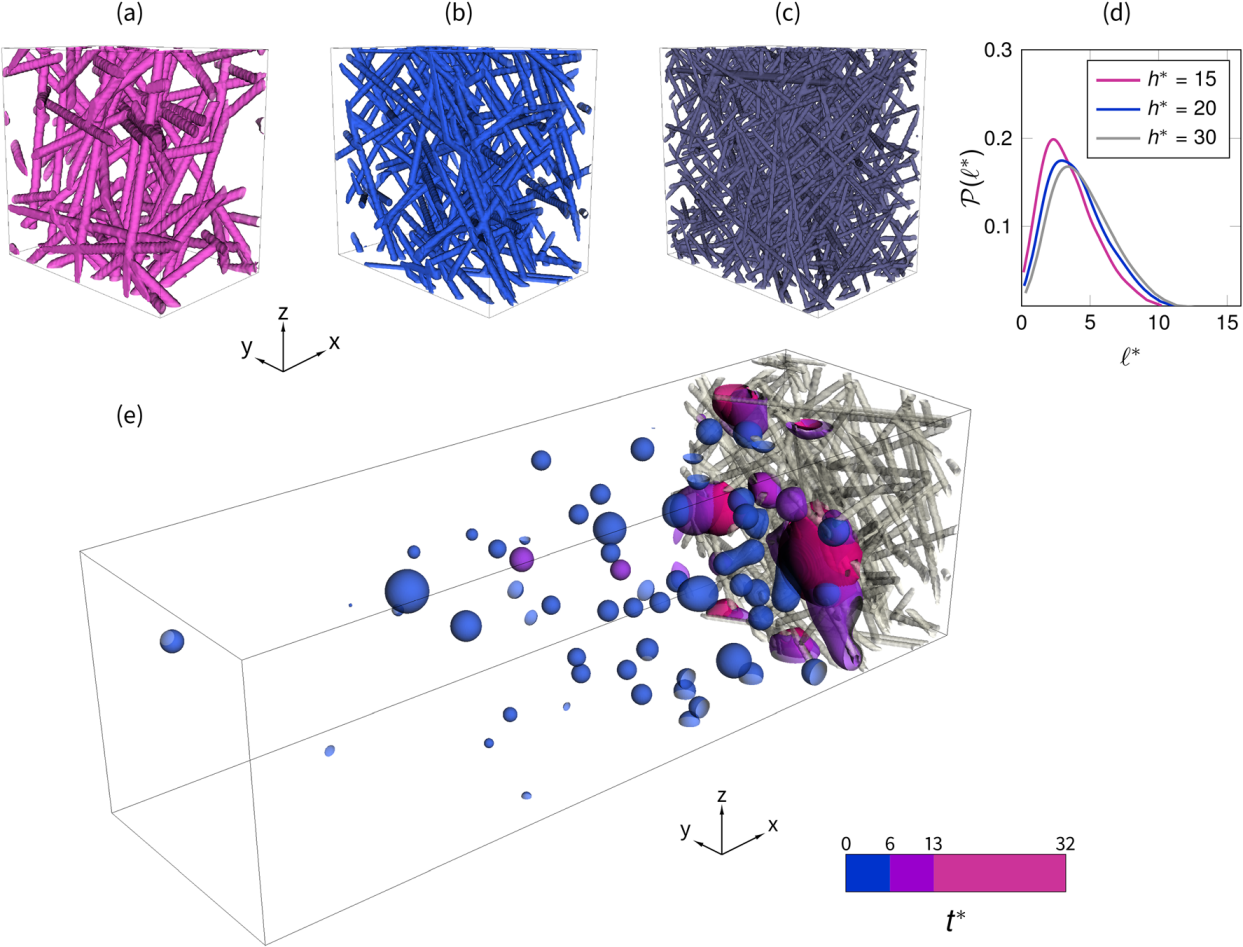
Three samples of artificially generated fibrous layers are showed for different thicknesses to the fiber size ratio (a) $h^{*}=15$, (b) $h^{*}=20$, and (c) $h^{*}=30$, at a fixed porosity ϵ=0.90, to which correspond $d_{0}^{*}=2,8/3,4$, respectively. Top right panel (d): the corresponding dimensionless pore size distributions $\ell^{*}=\ell /d_{f}$ along the in-plane directions *x* and *y* are similar, denoting the cross-sectional homogeneity of the samples. In the lower panel (e), we report the droplet distribution for one of the investigated cases at different dimensionless times $t^{*}=t/t_{0}$.

In Table I, we report the investigated values of *ϵ* and $h^{*}$, and in Figs. 1(a)–1(c), we show an example of such samples with different fiber sizes for the same porosity value. By computing the equivalent pore diameters along different cross sections on the planes (*x*, *y*) and (*x*, *z*) normal to the main fiber orientations, Fig. 1(d), we confirm that the dimensionless pore dimensions $\ell^{*}=\ell /d_{f}$ show minor differences at a fixed porosity (with ℓ being the pore size), meaning that the pore size statistic is rather homogeneous also along the through-plane thickness for $\sqrt{A}/d_{f}\geq 22.5$ and $h^{*}\geq 15$. To increase the statistical confidence, for each of the case reported in Table I, we generate four different random realizations and, when performing the analysis of data, we average between the results obtained for the different random realizations.

**TABLE I.** Investigated cases: microstructural parameters, porosity *ϵ*, thickness to fiber size ratio $h^{*}$, and impinging droplet-to-fiber size ratio $d_{0}^{*}$.

*ϵ*	$h^{*}$	$d_{0}^{*}$	*ϵ*	$h^{*}$	$d_{0}^{*}$	*ϵ*	$h^{*}$	$d_{0}^{*}$
0.92	15	2	0.80	15	2	0.90	30	4
0.90	15	2	0.90	20	8/3	0.86	30	4
0.86	15	2	0.86	20	8/3	0.80	30	4

### B. Fluid-dynamic conditions of incoming droplets

As previously discussed, the size of a droplet approaching a face mask fibrous layer can vary several orders of magnitude. In this study, we focus on droplets with sizes comparable to the fiber size. We place for each of the four random realizations 80 droplets of non-dimensional sizes $d_{0}^{*}=d_{0}/d_{f}=2,8/3,4$ randomly distributed in the proximity of the fibrous media within a three-dimensional box $V_{1}=A\;4h$. This choice allows us to avoid the complicated construction of spray-like inlet boundary conditions, whereas simple periodic boundary conditions are applied along the streamwise direction *x*. Given the typical size of fibers in fibrous layers, such dimensions correspond to droplets of size 10–40 μm, below the typical critical droplet size (the largest size of a droplet that does not fall within the life of the droplet) indicated in classic studies as 100 μm.^60^

To characterize the droplet dynamics and interception, we make use of the droplet Weber number

$$We=\frac{\rho u_{0}^{2}d_{0}}{\sigma },$$ (6)

as a measure of the inertial over surface tension forces, where *ρ* is the droplet density, *u*_0_ is the incident droplet velocity, and *σ* is the two-phase droplet-air surface tension. The incident velocity can vary greatly, depending on the flow direction (inhaling/exhaling), droplet size, distance from the source, and the type of expiratory event. If we take as a reference the more frequent expiratory events, talking and breathing, droplets velocities can often exceed 5 m/s. For water droplets of sizes 10–40 μm, with 1–5 m/s incident velocity, the Weber number results We∼O (0.1–10). From this estimation, one can evaluate the Stokes number for droplets of such a size range as $St=We\;\sigma /(18\mu_{2}u_{0})\;d_{0}^{*}\approx O(10–10^{3})$, where *μ*_2_ indicates the dynamic viscosity of the carrying fluid (air). Given that St≫1, we expect interception to be the dominant mechanism of filtration with droplets tending to follow their own trajectories rather than the one of the carrying fluid.

Through Eqs. (1) and (3), we apply an equivalent body force to the system. Such a force accelerates the droplets until they reach the fibrous layer, see, e.g., Fig. 1(e). On average, the droplets accelerate with an acceleration ΔP/L/ρ for a length 2*h* (half of the domain where the droplets are placed), which leads us to estimate the incident velocity as $u_{0}\approx \sqrt{4h\Delta P/L/\rho }$. We, thus, set the forcing term in order to get a value of the characteristic droplet Weber number We=0.5, to simulate expiratory events such as talking or breathing. In the present simulations, to this value of the Weber number corresponds a range of Stokes numbers St≈11–22, according to the droplet to fiber dimension $d_{0}^{*}=2–4$.

We then follow the dynamics of a total of 320 droplets per investigated case impinging on the fibrous layers. An example of such simulations is depicted in Fig. 1, where the droplets' spatial distribution is depicted at different dimensionless times $t^{*}=t/t_{0}$, where *t* is the simulation time and $t_{0}=\sqrt{\rho d_{0}^{3}/\sigma }$ is the impinging droplet characteristic time, based on the Weber number (spreading time). The two-phase viscosity ratio is here set as $\mu_{1}/\mu_{2}\approx 20$, where the indexes 1 and 2 indicate the viscous and less viscous phases (e.g., water and air), respectively. The simulations are stopped at $t^{*}=32$, a time that we found sufficient to intercept a relevant amount of droplets in the fibrous medium. Indeed, we will see that in such conditions, the fibrous layers are sufficiently thick to block 99% of the simulated droplets.

A total number of 24 simulations is performed, each of which is executed in parallel distributing the spatial domain on multiple cores. The lbdm^45^ algorithm exhibits a good parallel scaling, up to 15 000 cores. The computational cost for each simulation is around 100 core-hours, which highlights the efficiency of such a computational methodology and the possibility of extending the present study to larger domains and to further geometrical and fluid-dynamic parameters in future works.

## III. RESULTS

### A. Droplet penetration and medium permeability

**FIG. 2.**
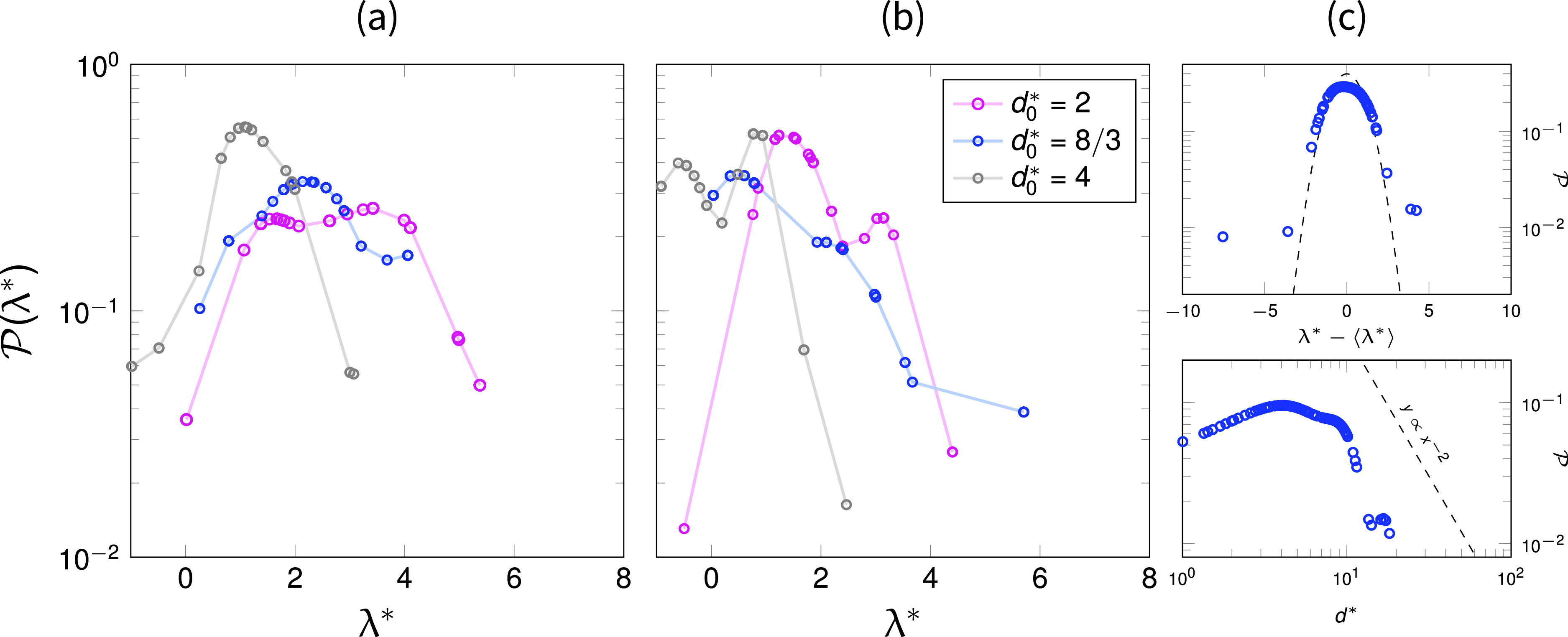
Volume-weighted probabilities of dimensionless droplet interception lengths $\lambda^{*}$ for (a) ϵ=0.90 and (b) ϵ=0.86. For each of the reported cases of porosities, we report the probabilities for different droplet-to-fiber size ratios $d_{0}^{*}$. (c) Upper panel, volume-weighted probabilities of droplet interception lengths shifted by their mean value, Eq. (7), which highlights a Gaussian distribution with unitary variance (the dashed line). Lower panel: probability distribution of dimensionless equivalent droplet diameters $d^{*}$ that shows a weak power-law decaying $d^{*}{}^{-2}$.

We first report the droplet positions within the medium after interception, at $t^{*}=32$, for six of the investigated cases. In Fig. 2(a), three cases with the porosity ϵ=0.9 and the droplet-to-fiber size ratio $d_{0}^{*}=2,8/3$, and 4 are shown, whereas in Fig. 2(b), the cases for the same values of $d_{0}^{*}$ are reported but for a lower value of porosity, ϵ=0.86. We do not observe any significant droplet breakup before impact, as expected given the imposed finite Weber number. Rather, we observe droplets merging and coalescing before and after entering the fibrous layer. To take into account such a phenomenon, we compute the volume-weighted probability distribution function of dimensionless interception lengths $\lambda^{*}=\lambda /d_{f}$, where *λ* is the streamwise distance traveled inside the porous medium by a droplet before interception. Thus, for a droplet sizing $2{\left(d/d_{0}\right)}^{3}$ (with *d* being the equivalent droplet diameter), we calculate a double probability of interception since that volume is the result of two droplets intercepted at close positions and successively coalesced. We observe, from Fig. 2, that droplet interception occurs deeper in the medium, as we increase the porosity (left panel) or we decrease the droplet-to-fiber size ratio (magenta marks), as expected.

For each of the investigated cases, we also compute the droplet mean interception length $\left\langle \lambda^{*}\right\rangle$, where again we make use of a volume-weighted formulation

$$\langle \lambda^{*}\rangle =\frac{\sum d^{*}{}^{3}\lambda^{*}}{\sum d^{*}{}^{3}},$$ (7)

where we sum over the observed droplet positions at the final instant $t^{*}=32$ and with $d^{*}=d/d_{f}$ we measure the dimensionless droplet size. It is worth highlighting here that at such an instant, we find the 96% of the droplets with a velocity of an order of magnitude lower than the incident velocity *u*_0_. This observation indicates that, even if mass transport can still be observed, its magnitude is much lower than the one experienced by droplets before impaction, and it must, thus, be considered a form of capillary transport occurring at a later stage after interception. In the only case of ϵ=0.92 and $d_{0}^{*}=2$, we notice a non-negligible transport within the medium for the 4% of the total droplet volume.

When we shift the droplet interception length $\lambda^{*}$ by their averaged values, Fig. 2(c) the upper panel, we observe that the probability of finding droplets in the vicinity of the mean droplet position appears Gaussian-distributed with unitary variance. This observation is evidencing that the dimensionless mean interception length $\left\langle \lambda^{*}\right\rangle$ is an important measure of the statistics of the traveled distances, around which the droplets spatially redistribute proportionally to the fiber size. (We remind that $\lambda^{*}=\lambda /d_{f}$.) In particular, roughly 95% of the droplets here simulated are found to be distributed at a distance from the mean interception length within two fiber sizes. However, we also have to consider this prediction as non-conservative, given the low value of the incoming droplet concentration; for a larger number of incoming droplets, one may expect a wider distribution. In the lower panel of Fig. 2(c), we also report the probability of equivalent droplet diameters $d^{*}$ within the media, which shows the great number of coalescing events that increases the average droplet diameters, $d_{0}^{*}>1$, and a weak power-law decaying, $d_{0}^{*-2}$ suggesting a possible Pareto distribution. (A Pareto distribution has also been suggested to describe respiratory droplet size distribution in coughs.^61^)

**FIG. 3.**
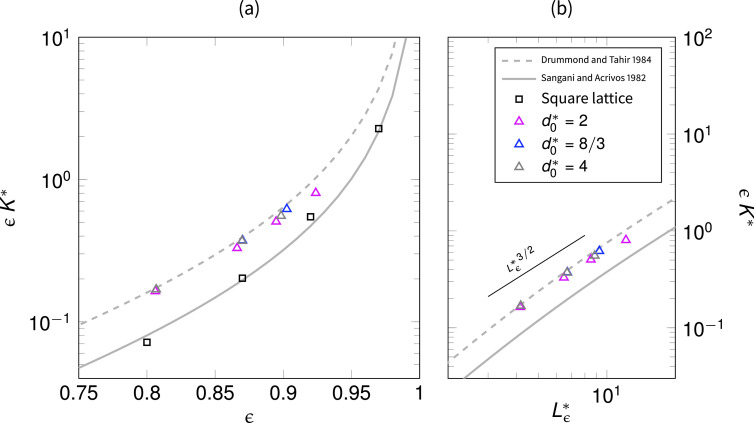
(a) Darcian permeabilities $\epsilon K^{*}$ as a function of porosity *ϵ*, where we report also the analytical solutions for fibers aligned perpendicular (solid line) and parallel (dashed line) to the flow. A computation of permeabilities for perpendicular fibers regularly placed in a square lattice is showed with squared marks for model validation and comparison, indicating an excellent agreement with the theory. On the right panel (b), the power-law relationship between Darcian permeabilities and diffusive intercept lengths $L_{\epsilon }^{*}$ is showed with a proportionality $\epsilon K^{*}\propto L_{\epsilon }^{*}{}^{3/2}$.

To complete the picture regarding the efficiency of fibrous layers, together with their efficacy in intercepting incoming droplets, we have also to consider the comfort that such layers provide to the users. Such a comfort is strongly dictated by the layer breathability, which can be measured via the computation of the medium permeability. Indeed, highly permeable media would allow a low pressure drop in the air flow and easy breathing, but at the cost of low interception efficiency. We compute the permeability with the simulation of single phase flow though the triperiodic fibrous structures, as the one, e.g., depicted in Fig. 1. The permeability is calculated through Darcy's law as

$$K^{*}=\frac{u_{2}\mu_{2}}{d_{f}^{2}}{\left(\frac{\Delta P}{L}\right)}^{-1},$$ (8)

where *u*_2_ indicates the less viscous phase (e.g., air) intrinsic velocity. In Fig. 3(a), we report the measured Darcian permeabilities $\epsilon K^{*}$ as a function of the porosity. Interestingly, when compared with the analytical solutions for transversally^62^ and streamwise^63^ aligned fibrous media, we found that the fibrous layers exhibit permeabilities comparable with the latter solution, indicating that the mild orientation of the fibers along the flow direction has a great effect on the resistance. We also here stress that face masks are usually composed of multiple layers, and thus, the individual layer permeability here computed can be affected by the presence of transition zones between layers with different porosities and microstructures, but also between zones of free flows (airflow over the mask surface) and the porous region (the mask itself).^64^

We also notice, from Fig. 3(b), that the computed layer permeabilities show a power-law dependence with the microstructural parameter $L_{\epsilon }^{*}$, which is the ratio between the so-called mean geometrical intercept length (where the adjective geometrical is used here for discerning it from the droplet mean interception length $\left\langle \lambda^{*}\right\rangle$), defined via the specific surface area *S_s_*, and the fiber size *d_f_* as

$$L_{\epsilon }^{*}=\frac{4\epsilon }{S_{s}}\frac{1}{d_{f}}.$$ (9)

For fibrous media, the specific surface area $S_{s}=\pi d_{f}/\ell_{c}^{2}$, with $\ell_{c}$ being the characteristic pore size length. The latter can be reformulated as $\ell_{c}^{2}=\pi /4d_{f}^{2}/(1-\epsilon )$ to obtain $L_{\epsilon }^{*}=\epsilon /(1-\epsilon )$, which is indeed a well-known microstructural parameter in the formulation of permeability predictions such as the Kozeny–Carman equation. The mean geometrical intercept length is defined as the mean length of the segments of a randomly drawn line that lie in the void space, and thus, it is also a measure of a characteristic pore size.^65^ In Sec. III B, we will see that this length has an important meaning in the characterization of the droplet interception ability of a fibrous porous medium. We will make use of microstructural parameter $L_{\epsilon }^{*}$ and of the droplet-to-fiber size ratio $d_{0}^{*}$ to formulate a model for the prediction of droplets interception in fibrous layers.

### B. Small, large, and medium-sized pores

**FIG. 4.**
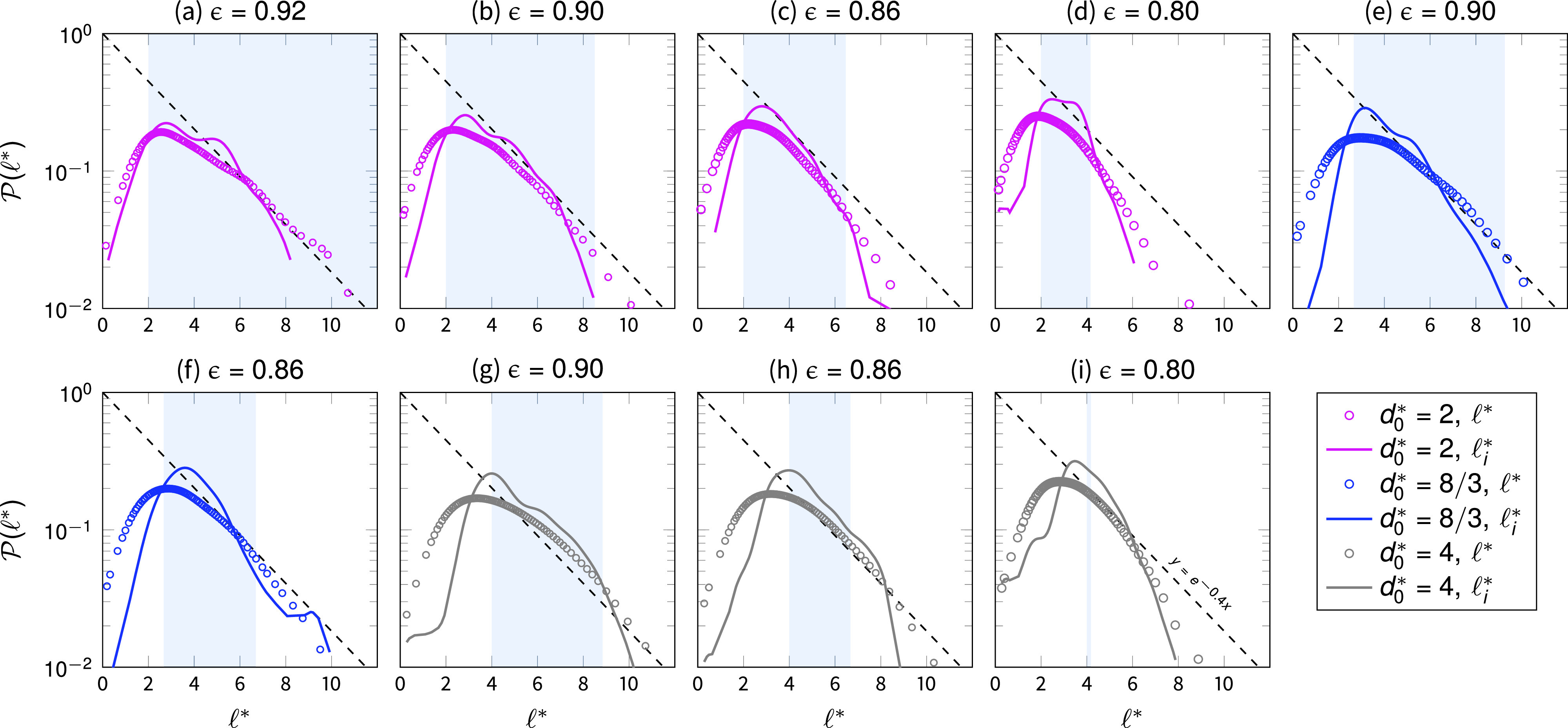
Pore size distributions for all the available pores $\ell^{*}$ (circle marks) and invaded pores $\ell_{i}^{*}$ (solid lines), categorized according to the different porosity values, panels (a)–(i) from the top left to the right bottom, and droplet-to-fiber size ratio. The shaded area indicates the interval of the integral in Eq. (13).

With the aim of formulating a predictive model for droplet interception, based on the observed numerical results, we argue that the distance that a droplet travels before interception, on average, depends first on the probable size of the pore that the incoming droplet encounters when entering the fibrous medium. Such a probability is related to the pore size distribution of the medium. In Fig. 4, we have reported the pore size distributions $P(\ell^{*})$ for each of the investigated media (circle marks), where with $\ell^{*}=\ell /d_{f}$, we indicate the dimensionless pore size. The pore size distribution has been computed with a two-dimensional water shed algorithm in different cross sections of each sample, belonging to the two-dimensional planes normal to the main fiber orientations, (*x*, *y*) and (*x*, *z*). We notice an exponential decay of such a probability as the size of the pores increases, till approximately 12 fiber sizes.

Along with the pore size distributions, we also compute the probability density functions of the invaded pore sizes $\ell_{i}^{*}$ at $t^{*}=32$ (solid lines). The invaded pores are defined as the pores completely filled with the more viscous phase or containing at least half of a droplet volume within their pore subspace farther from the medium inlet. By defining this subspace as the pore space lying within $\ell_{x}^{*}-d_{0}^{*}$ and $\ell_{x}^{*}$, with $\ell_{x}^{*}$ being the streamwise length of the pore, we ensure to address as invaded the pores within which a droplet had traveled a distance comparable with the pore length and had later being intercepted. Compared with the medium pore size distribution, the probability of the invaded pores $P(\ell_{i}^{*})$ exhibits a more-narrow shape for all the considered cases, indicating that small and large pores are less prone to be invaded. In particular, we notice that $P(\ell_{i}^{*})$ shows a rather sharp decay for pore sizes for which $\ell^{*}<d_{0}^{*}$ and $\ell^{*}>L_{\epsilon }^{*}$.

On the basis of these observations, we can define three types of pores: (i) the small pores, for which $\ell^{*}\leq d_{0}^{*}$, (ii) the medium-sized pores, for which $d_{0}^{*}<\ell^{*}\leq L_{\epsilon }^{*}$, and (iii) the large pores with sizes $\ell^{*}>L_{\epsilon }^{*}$. The small pores exhibit a size smaller than the droplet size; thus, if encountered, they should not be easily accessible given the high capillary pressure necessary for their invasion. We argue that small pores provide, thus, very little room for transport, explaining their observed low probability of invasion. The medium-size pores are instead more accessible, and their size should allow droplet invasion and successive collection.

While for small and medium-size pores, these arguments are intuitive, less clear is the reason behind the observed low probability of droplet invasion within the large pores. By looking at the dynamics of pore invasion, we observe that large pores are often surrounded by a relative high number of fibers. A high number of fibers placed along the pore perimeter means a significant amount of surface for droplet collection and, on average, a small pore throat size to access the pore space, which should be an important parameter to define the probability of invasion of a pore. We, thus, compute the pore-scale specific surface area *S_p_*, to determine the amount of fiber surface along the pore perimeter relative to the pore size. Similarly, to the computation of pore size distribution, we compute *S_p_* for each pore identified in different cross sections along the two-dimensional planes (*x*, *y*) and (*x*, *z*). We find that, for sufficiently large pores, the computed value of *S_p_* is constant for each sample, meaning that, as the pores get larger, a higher number of fibers are encountered along their perimeter. Such a concept should be indeed a direct consequence of the homogeneity of the medium for sufficiently large representative elementary volumes. We here stress that *S_p_* is the specific surface related to each individual pore space, and it is, thus, a microscopic quantity, unlike *S_s_* used in Eq. (9) to calculate $L_{\epsilon }^{*}$, which is instead based on the macroscopic parameter *ϵ*.

**FIG. 5.**
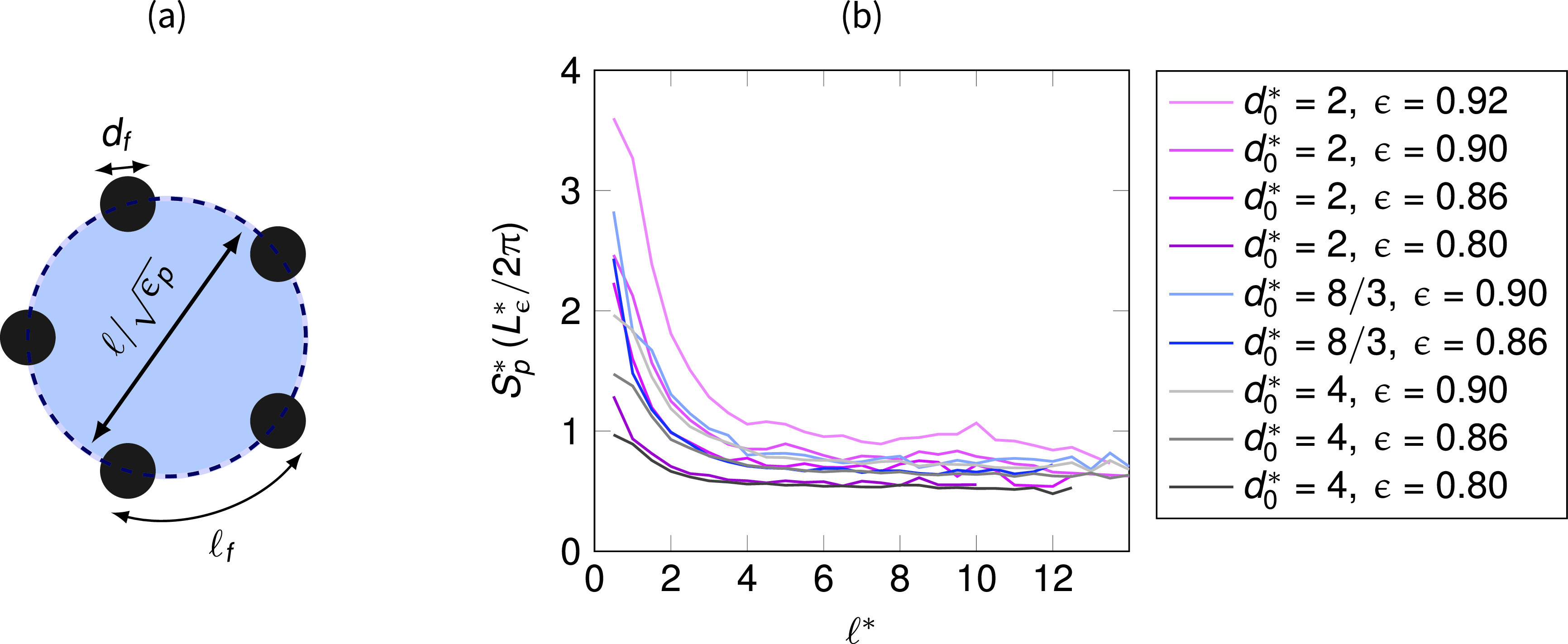
(a) Sketch representing the microscopic pore space characterized by a pore area $\pi \ell^{2}/4$ and delimited by *n_f_* different fibers of diameter *d_f_*. The microscopic porosity is *ϵ_p_*, while $\ell_{f}$ indicates the average distance between fibers. The pore-scale specific surface area *S_p_* derived in Eq. (10) is given by the ratio between the sum of half-perimeter of the fibers $n_{f}\pi d_{f}/2$ and the pore space $\pi \ell^{2}/4$. In the right panel (b), we report the computation of the factor $S_{p}^{*}L_{\epsilon }^{*}/(2\pi )\to 1$ for each investigated case.

To interpret this result, let us take as an example a two-dimensional circular pore of size $\pi \ell^{2}/4$, enclosed between *n_f_* circular fibers with diameter *d_f_*, such as the one depicted in Fig. 5. The dimensionless pore-scale specific surface area $S_{p}^{*}=S_{p}\;d_{f}$ for this case reads as

$$S_{p}^{*}=\frac{2n_{f}}{\ell^{*}{}^{2}},$$ (10)

and we can define the average distance between fibers as

$$\ell_{f}^{*}∼\frac{\pi \ell^{*}}{\sqrt{\epsilon_{p}}n_{f}}=\frac{2\pi }{S_{p}^{*}}\frac{1}{\sqrt{\epsilon_{p}}\ell^{*}}\;.$$ (11)

Finally, we write the average pore throat size between fibers placed along the pore perimeter of such a pore in the dimensionless form as

$$\ell_{f}^{*}-1∼(\frac{2\pi }{L_{\epsilon }^{*}S_{p}^{*}})\frac{L_{\epsilon }^{*}}{\ell^{*}}\frac{1}{\sqrt{\epsilon_{p}}}-1,$$ (12)

with *ϵ_p_*, the pore-scale local porosity. Equations (10)–(12) are visually represented in the sketch of Fig. 5. In the right panel of Fig. 5, we report the computation of the factor $2\pi /(L_{\epsilon }^{*}S_{p}^{*})$, under brackets on the right-hand side of Eq. (12), performed in our fibrous samples. The factor approaches unity, i.e., $S_{p}L_{\epsilon }^{*}/(2\pi )∼1$. This observation gives us the opportunity to directly interpret Eq. (12): since for large pores we expect $\epsilon_{p}\to 1$, we can estimate the pore-throat-to-fiber size ratio as $\ell_{f}^{*}-1∼L_{\epsilon }^{*}/\ell^{*}-1$ and find that such a size is a positive value as long as $\ell^{*}<L_{\epsilon }^{*}$. In other words, pores of the size larger than $L_{\epsilon }^{*}$ are accessible through small if not fully occluded pore throats.

**FIG. 6.**
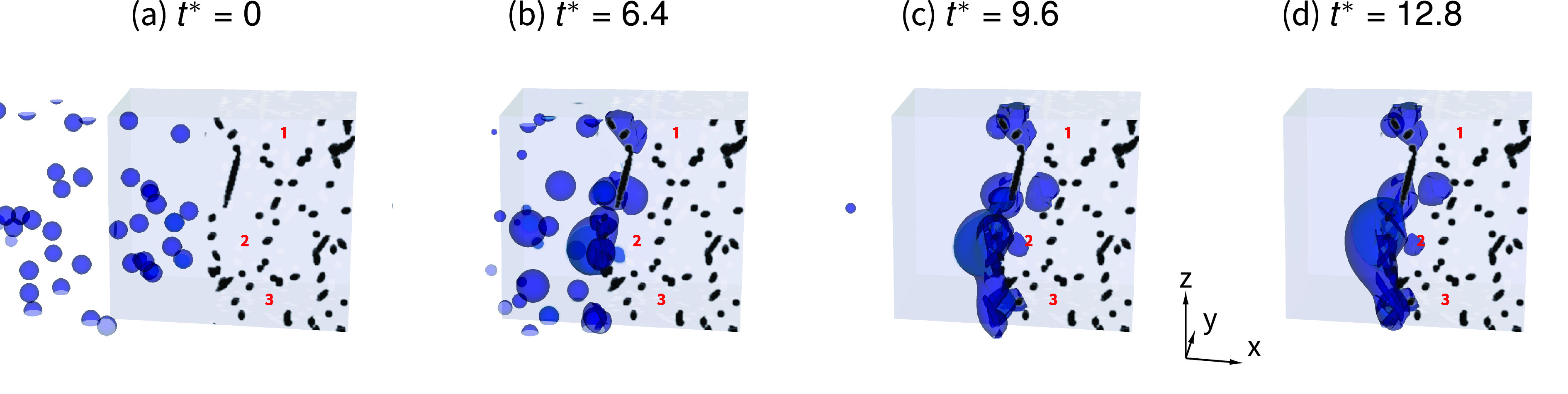
Snapshots of droplets intercepted in a fibrous medium with ϵ=0.86 and $d_{0}^{*}=8/3$, at different dimensionless times, (a) $t^{*}=0$, (b) $t^{*}=6.4$, (c) $t^{*}=9.6$, and (d) $t^{*}=12.8$. Three pores of a large size are indicated with the numbers 1, 2, and 3. The droplets accumulate at the small pore throats laying along the large pore perimeters, coalescing, and impeding the access to the large pores. Eventually, of the three large pores, only pore number 2 is invaded.

The droplet invasion in large pores is, thus, frequently impeded by the limited size of the pore throats found along the pore perimeter, where such constrictions and a high number of fibers collect the droplets, which coalescence further blocking the access to large pores. An example of such a mechanism is depicted in Fig. 6, where we show that the probability of droplet invasion in large pores is conditioned by the significant coalescing phenomena occurring at the small pore throats. This observation is in line with the significant increase in the equivalent droplet diameters reported in Fig. 2(c).

### C. A model for droplet interception in fibrous porous media

Summarizing, we observe that the large majority of the droplets are collected within medium-size pores. Small pores, for which $\ell^{*}<d_{0}^{*}$, are less prone to be invaded given their small pore to droplet size, while the access to large pores, for $\ell^{*}>L_{\epsilon }^{*}$, is often inhibited at their entrance by the limited size of the pole throats. In the interception regime, at finite Weber and high Stokes numbers, when accessing a medium-size pore, a droplet travels approximately the pore length and gets intercepted at its ending pore subspace, collected by one or more fibers. Following this reasoning, we can approximate the droplet mean interception length as

$$\langle \lambda^{*}\rangle ∼\int_{d_{0}^{*}}^{L_{\epsilon }^{*}}P(\ell_{i}^{*})\ell_{i}^{*}d\ell_{i}^{*}-\beta ,$$ (13)

where we assume a negligible effect on the invasion dynamics of the small and large pores as defined above.

The lower limit of the integral indicates the limiting sizes for medium-size pores, i.e., $d_{0}^{*}<\ell^{*}<L_{\epsilon }^{*}$. When a droplet is collected by fibers, it can clog a pore entrance and any successive incoming droplet should accumulate at such a position. Such an accumulation should shift the droplet mean interception length toward the inlet of the medium, and it is accounted in the formulation of Eq. (13) through the parameter *β*. In the limit case for which the droplet size is comparable with the size of the largest of the medium-sized pores, i.e., for $d_{0}^{*}∼L_{\epsilon }^{*}$, the droplet penetration within the medium is greatly limited since the droplets are larger than the most probable sites of invasion (the medium-sized pores). The infiltration occurs mainly through droplets squeezing and capillary transport, while a large amount of droplets volume accumulates at the pores entrance and medium inlet. In this situation, Eq. (13) predicts a negative value of the droplet mean interception length, which indicates such an accumulation: the integral of the right-hand side vanishes and $\langle \lambda^{*}\rangle ∼-\beta <0$.

**FIG. 7.**
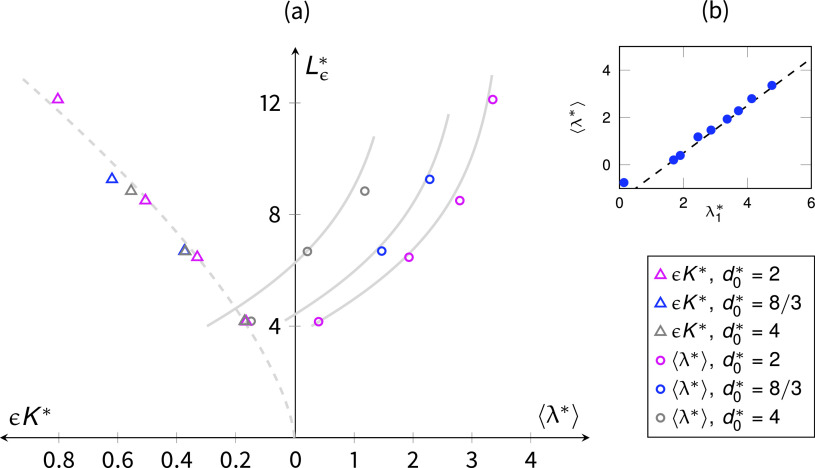
(a) Efficiency of the fibrous layers, in terms of Darcian permeability $\epsilon K^{*}$ (left x-axis) and the droplet mean interception length $\left\langle \lambda^{*}\right\rangle$ (right x-axis), as a function of the microstructural parameter $L_{\epsilon }^{*}$ (y-axis) and the droplet-to-fiber size ratio $d_{0}^{*}$. As we increase the porosity and $L_{\epsilon }^{*}$ of a layer, we observe a superlinear increase in the breathability $\epsilon K^{*}\approx 0.02\;L_{\epsilon }^{*}{}^{3/2}$ (dashed line) and a sublinear increase in the average length traveled by droplets $\left\langle \lambda^{*}\right\rangle$, expressed by the model of Eq. (14) for the different values of $d_{0}^{*}$ (solid lines). In the top-right panel (b), we report $\left\langle \lambda^{*}\right\rangle$ as a function of the first term of Eq. (14), for *a* = 0.4, indicated as $\lambda_{1}^{*}$. The dashed line shows the solution y=x−β, with β=3/2.

To solve Eq. (13), we need to estimate the probability distribution of invaded pore sizes within the fibrous medium. In Fig. 4, we have reported such a distribution for all the considered cases, where we also indicate, with shaded areas, the integral interval of interest for solving Eq. (13). We note that the invaded pore size distributions $P(\ell_{i}^{*})$ are well approximated by an exponential function, and we can then reformulate Eq. (13) as

$$\begin{array}{c} \langle \lambda^{*}\rangle ∼\int_{d_{0}^{*}}^{L_{\epsilon }^{*}}e^{-a\ell^{*}}\ell^{*}d\ell^{*}-\beta ={\left[\frac{e^{-a\ell^{*}}}{a^{2}}(a\ell^{*}+1)\right]}_{L_{\epsilon }^{*}}^{d_{0}^{*}}-\beta , \end{array}$$ (14)

where the parameter *a* denotes the magnitude of the exponential decay characterizing the invaded pore size distribution of the medium. By applying Eq. (14) to predict the droplet mean interception length, we find an excellent agreement with the parameter *a* = 0.4 and β=3/2, reported in Fig. 7(b). We observe indeed that the invaded pore size distributions show an exponential right tail $P(d_{0}^{*}<\ell_{i}<L_{\epsilon }^{*})\approx \;\text{exp}\;(-a\ell_{i}^{*})$ within the interval of medium-sized pores. The value of β=3/2 points out that the accumulation of droplets occurs within approximately three halves fibers from the pore throats that give access to the pore spaces.

In Fig. 7(a), we graphically summarize the results. The efficiency of the fibrous layer is measured in terms of breathability (Darcian permeability $\epsilon K^{*}$) and interception efficiency with the lower values of $\left\langle \lambda^{*}\right\rangle$ indicating a higher efficiency. We observe that such an efficiency depends mainly on two parameters, the microstructural parameter $L_{\epsilon }^{*}$, a function of porosity, and the droplet-to-fiber size ratio $d_{0}^{*}$. By increasing the porosity, one obtains a superlinear growth of the breathability, proportional to $L_{\epsilon }^{*}{}^{3/2}$ (the left axis). At the same time, the growth of the droplets averaged traveled distance before interception results sublinear, as Eq. (14) predicts. Such an observation suggests that an improvement in the breathability could cost little in terms of droplet penetration, for ranges of porosity that correspond to $8<L_{\epsilon }^{*}<12$.

We also point out that one could theoretically try to design an optimal fibrous layer by increasing the relative number of large pores $\ell^{*}>L_{\epsilon }^{*}$ at the expense of the medium-sized ones, in order to possibly increase the permeability without compromising interception efficiency. However, to confirm the efficacy of such a design strategy, a measure of the effective increase in permeability with this specific pore configuration is demanded in future works and, at the moment, it is still elusive to us.

One has to keep in mind that, from a design perspective, the thickness of the layer relative to the fiber size should be much larger than the porosity-dependent microstructural parameter, i.e., $h^{*}\gg L_{\epsilon }^{*}$. Equation (13) predicts that the majority of the droplets is collected within a distance $L_{\epsilon }^{*}$. However, we observe a small amount of droplets traveling for longer distances within the medium. Based on the observed probabilities for pore invasion, a rough approximation for $\ell_{i}^{*}>L_{\epsilon }^{*}$ can read as $P(\ell_{i}^{*}>L_{\epsilon }^{*})\approx \;\text{exp}\;(-\ell_{i}^{*}+(1-a)L_{\epsilon }^{*})$, which denotes the fast exponential decay of the invasion probability for large pores. One can, thus, estimate the filter medium collection efficiency for layers whose thickness $h^{*}$ is larger than $L_{\epsilon }^{*}$ as $\eta \approx 1-\text{exp}\;(-h^{*}+(1-a)L_{\epsilon }^{*})$. Through this estimation, we obtain η>99.9% for all the considered cases, in line with the observed high collection efficiency of the fibrous media here investigated.

## IV. CONCLUSIONS

We have presented results of pore-scale direct numerical simulations of droplets interception in fibrous porous media that compose face masks. In particular, we focus our analysis to droplet-to-fiber size ratios within the range $d_{0}^{*}=2–4$ to represent respiratory droplets generated during talking or breathing. During these expiratory events, we simulate droplets of the size of 10–40 μm that exhibit high traveling velocities, characterized by a finite and a high value of the Weber and Stokes numbers, respectively, and whose mechanism of collection in a porous medium is mainly interception.

By analyzing the statistics of droplet trajectories before impaction with fibers and the probability of droplets invasion within the fibrous microstructure, we have identified three categories of pores: (i) small pores of the size smaller than the mean droplet diameter, $\ell^{*}<d_{0}^{*}$, (ii) large pores of the size larger than the microstructural parameter $L_{\epsilon }^{*}<\ell^{*}$, and (iii) medium-sized pores, for which the pore size is placed within the range $d_{0}^{*}<\ell^{*}<L_{\epsilon }^{*}$. We find that the droplets tend to invade and be collected in the latter category of pores, while, counterintuitively, larger pores do not provide an easy access to droplets. We argue that this occurs because large pores are delimited by a high number of fibers along their perimeter, which ultimately reduces the pore-throat size in their proximity. Coalescence is further enhancing this phenomenon, because droplets tend to accumulate at these small pore-throat sizes, limiting even to a greater extent the access to the large pores.

We reason that, for a given droplet-to-fiber size ratio $d_{0}/d_{f}$, the efficiency of a fibrous layer composing a mask, in terms of respiratory droplets interception and breathability, can be estimated via the microstructural parameter $L_{\epsilon }^{*}=\epsilon /(1-\epsilon )$, a function of the porosity *ϵ*. We indeed observe a power-law scaling of the Darcian permeability with the same microstructural parameter, denoting a superlinear growth, i.e., $\epsilon K^{*}\propto L_{\epsilon }^{*}{}^{3/2}$. Furthermore, we propose a model to predict the average distance traveled by droplets within the fibrous porous media before interception (the mean interception length), based on the values of $d_{0}^{*}$ and $L_{\epsilon }^{*}$, which exhibit a sublinear growth with $L_{\epsilon }^{*}$. Finally, we discuss possible design improvements of fibrous layers in face masks inspired by the above observations. In particular, we stress the importance of designing fibrous layers whose thickness is larger than the value $L_{\epsilon }^{*}d_{f}$, since we have seen that the majority of the droplets are intercepted within this distance. In addition, given the superlinear and sublinear growths of permeability and droplet penetration, respectively, we note that a possible improvement in breathability, for high values of porosity ϵ≥0.9, should cost little in terms of filtration efficiency.

In the perspective of extending the present study to cover further aspects related to filtration efficiency in the fibrous porous media, future research activities could explore the dynamics of smaller and polydisperse droplets in order to better represent realistic expiratory events. To this end, different incident droplet velocities could also be simulated, in order to represent, e.g., more extreme expiratory events such as sneezing. Given the efficiency of the numerical algorithm, larger porous domains could also be simulated, possibly representing hydrophobic/hydrophilic multilayer structures such as the one composing 3ply disposable face masks, which induce porosity and permeability gradients along the flow directions that strongly affect two-phase flow behaviors.^43,66^ Nevertheless, to simulate droplets smaller than 1 *μ*m, where diffusion is the main filtration mechanisms, hybrid lattice-Boltzmann–Lagrangian-based methods could be preferred in order to solve scales smaller than the carrying flow resolution for the droplets transport and limit the computational cost.^67^ To reconstruct the microstructure via non-destructive measurement techniques, such as x-ray computed tomography, is another interesting approach to access geometrical information of more realistic geometries, possibly identifying the effects of degradation of the materials.^68,69^ Finally, the non-Newtonian behavior of the liquid droplets should be modeled to catch possible deviation from the fluid-dynamic Newtonian behavior of the collected droplets.^42^

## Data Availability

The data that support the findings of this study are available from the corresponding author upon reasonable request.
